# COMBINED PLATE VERSUS EXTERNAL FIXATION FOR DISTAL RADIUS FRACTURES

**DOI:** 10.1590/1413-785220233101e252977

**Published:** 2023-04-17

**Authors:** Oktay Polat, Serdar Toy, Hakan Özbay

**Affiliations:** 1 Sultanbeyli State Hospital, Department of Orthopedics and Traumatology, İstanbul, Turkey.; 2 Basaksehir Pine and Sakura City Hospital, Department of Orthopedics and Traumatology, Istanbul, Turkey.; 3 Acıbadem Taksim Hospital, Department of Orthopedics and Traumatology, Istanbul, Turkey.

**Keywords:** Fractures, Bone, Radius Fractures, Fracture Fixation, Bone Plates, Fraturas Ósseas, Fraturas do Rádio, Fixação de Fratura, Placas Ósseas

## Abstract

**Objectives:**

This study aimed to compare the functional and radiological results of patients who had intra-articular comminuted distal radius fractures and were operated on with external fixation percutaneous pinning or the volar-dorsal combined plate osteosynthesis.

**Methods:**

In this study, 49 patients operated on and followed up for the comminuted distal radius fractures between May 2015 and January 2019 were retrospectively evaluated. The surgical outcomes of the patients, who were operated on with combined dorsal-volar plate osteosynthesis or external fixation percutaneous pinning, were compared in this study. Functional and radiological scores were evaluated and analyzed statistically.

**Results:**

There was no statistical difference between external fixation and volar-dorsal combined plate groups regarding the Disabilities of the Arm, Shoulder, and Hand (DASH) questionnaire, the Visual Analog Scale (VAS), the Mayo scoring system, range of motion, and grip strength values.

**Discussion:**

Although the combined volar-dorsal plate osteosynthesis technique had a longer operation time and a more complicated surgical procedure, the combined volar-dorsal plate osteosynthesis had lower complication rates and permitted early mobilization. The combined volar-dorsal plate osteosynthesis could be an alternative to external fixation percutaneous pinning. ***Level of Evidence III, Therapeutic Studies Investigating the Results of Treatment.***

## RESUMO

**Objetivo:**

O objetivo deste estudo foi comparar os resultados funcionais e radiológicos de pacientes portadores de fraturas intra-articulares cominutivas do rádio distal, operados com fixação externa percutânea ou com osteossíntese volar-dorsal de placa combinada.

**Método:**

Foram avaliados 49 pacientes operados e acompanhados por fraturas cominutivas distais do rádio entre maio de 2015 e janeiro de 2019 retrospectivamente. Os resultados cirúrgicos dos pacientes operados com osteossíntese de placa volar-dorsal combinada ou fixação externa percutânea foram comparados. Os escores funcionais e radiológicos foram avaliados e analisados estatisticamente.

**Resultados:**

Não houve diferença estatística entre os grupos de fixação externa e placa combinada volar-dorsal em relação ao questionário Deficiências do braço, ombro e mão (DASH), à Escala Visual Analógica (VAS), ao sistema de pontuação Mayo, à amplitude de movimento ou à avaliação de força de preensão.

**Conclusão:**

Embora a técnica combinada de osteossíntese da placa volar-dorsal tenha um tempo cirúrgico mais longo e procedimento mais complicado, a osteossíntese combinada da placa volar-dorsal apresentou menores taxas de complicações, permitindo a mobilização precoce. A osteossíntese combinada da placa volar-dorsal pode ser uma alternativa à fixação externa percutânea. **Nível de Evidência III, Estudos terapêuticos – Investigação dos resultados do tratamento.**

## INTRODUCTION

Distal radius fractures are among the most common fracture.^1^ Distal radius fractures account for 7.5% of all fractures and 15.7% of all upper extremity fractures.^2^ The functional outcome of distal radius fractures is affected by extra-articular alignment, anatomical reduction of the articular surface, intra-articular soft tissue injuries, and postoperative complications.^3-5^

Although the traditional treatment is direct reduction plastering, surgery is required for intra-articular and unstable fractures. Many surgical procedures are available. These are percutaneous pinning, external fixation, volar plating, and dorsal plating.^6-8^

Percutaneous pinning is mostly used for extra-articular fractures.^9-11^ The volar plating technique is preferred by most surgeons because of its relatively easy surgical approach, facilitating early motion initiation, and having fewer soft tissue complications than dorsal plating.^7^ However, it is challenging to fix dorsal parts with volar plates. Dorsal plates are used to fix dorsal parts, but the surgeon avoids dorsal plates due to the high incidence of complications such as extensor tendon irritation and rupture. In addition, the adaptation of the plate to the radius anatomy is difficult.^12^

External fixation neutralizes the pressure forces in the fracture area by applying distraction to the joint surface. External fixation is supported by percutaneous or mini-open methods. External fixation has been described as a treatment modality equally suitable for volar plating in dorsally displaced intra-articular distal radius fractures.^8-13^ Partial intra-articular fracture treatments may not be sufficient. Intra-articular fractures are displaced in more than one plane. Intraarticular comminuted fractures are mostly treated with percutaneous pinning complementary to external fixation.^14^ The disadvantage of this method is that the joint surface cannot be corrected sufficiently due to the fact that arthrotomy cannot be performed during the surgery, and the joint range of motion cannot be achieved due to the inability to give early motion.^3-6^

In this study, we aimed to compare the functional and radiological results of patients, who had intra-articular comminuted distal radius fractures, were operated on with external fixation percutaneous pinning or combined volar-dorsal plate. We, in this study, hypothesized that the dorsal-volar combined plate osteosynthesis might be superior to the external fixation percutaneous pinning for distal radius comminuted fractures in terms of clinical functionality.

## METHODS

### Design and Sample

In this study, patients who were operated on and followed up for the comminuted distal radius fractures between May 2015 and January 2019 were retrospectively evaluated. The study protocol was approved by the local ethics committee (IRB Date/No:28.01.2021/35). Written informed consent was obtained from each patient. The study was conducted in accordance with the principles of the Declaration of Helsinki.

The inclusion criteria were as follows: (1) patients were operated on with the combined dorsal-volar plating and external fixation to treat complex four-part distal radius fractures (shaft, radial styloid process, dorsal medial facet, and volar medial facet) between May 2015 and January 2019; (2) patients with at least two years of followup and regular follow-up. The exclusion criteria were as follows: (1) patients whose bone maturation was incomplete (n=2); (2) patients with additional injuries in the same extremity (n=3); (3) fractures extending into diaphysis (n=3); open and pathological fractures (n=2); (4) patients had previously undergone surgery on the same extremity (n=3). Five patients were excluded during the follow-up. Forty-nine patients (20 females, 29 males; mean age 43.85±15.78; range, 18 to 74 years) who met the criteria were included.

The patients were divided into two groups. Combined volar-dorsal plates (Group 1) were applied to 23 patients (46.9%). Twenty-six patients (53.1%) were operated on with external fixation (Group 2). The operation method was made according to the surgeon's own experience and preference. One surgeon applied the combined volar-dorsal plate for complex fractures, while another surgeon performed the external fixation.

### Surgical Procedures

**Figure 1:**
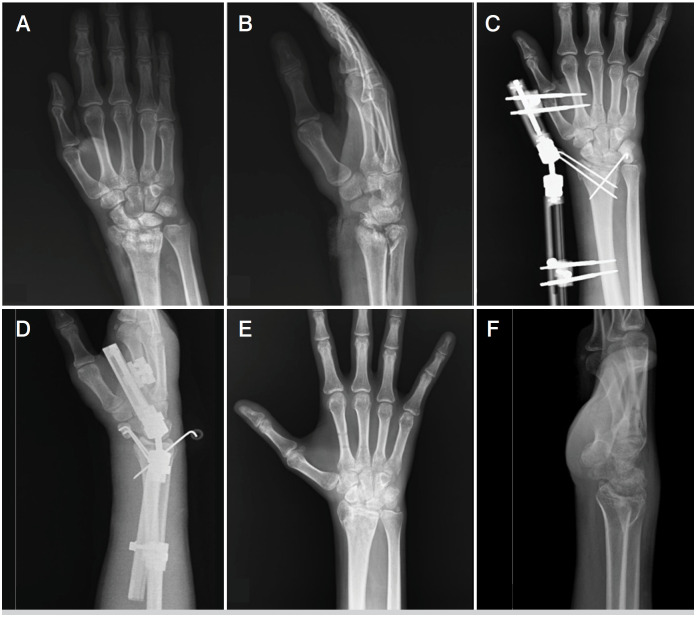
A) Preoperative anteroposterior radiograph showing distal radius fracture, distal radius fracture, AO 2R3-C3, Melone type 4. B) Preoperative lateral radiograph showing distal radius fracture, distal radius fracture, AO 2R3-C3, Melone type 4. C) Early postoperatively X-ray with anteroposterior after fixation with an external fixator. D) Early postoperatively X-ray with lateral after fixation with an external fixator. E) X-ray with anteroposterior after the implant removal. F) X-ray with lateral after the implant removal.

Two orthopedic surgeons performed all surgical procedures. Closed reduction was performed for external fixation using image intensification. Percutaneous K-wires or small elevators through a small incision for manipulation were used when complete reduction after closed reduction could not be achieved (Figure 1). Arthrotomy was not performed for any patients. K-wires were typically 1.6 mm in size. One uniplanar bridging external fixator (TST, Istanbul, Turkey) system was used. External fixations were removed in the outpatient clinic at 4 to 6 weeks. Then, all patients were given standardized physical therapy for two weeks. Then, home exercises were instructed five times a day.

**Figure 2:**
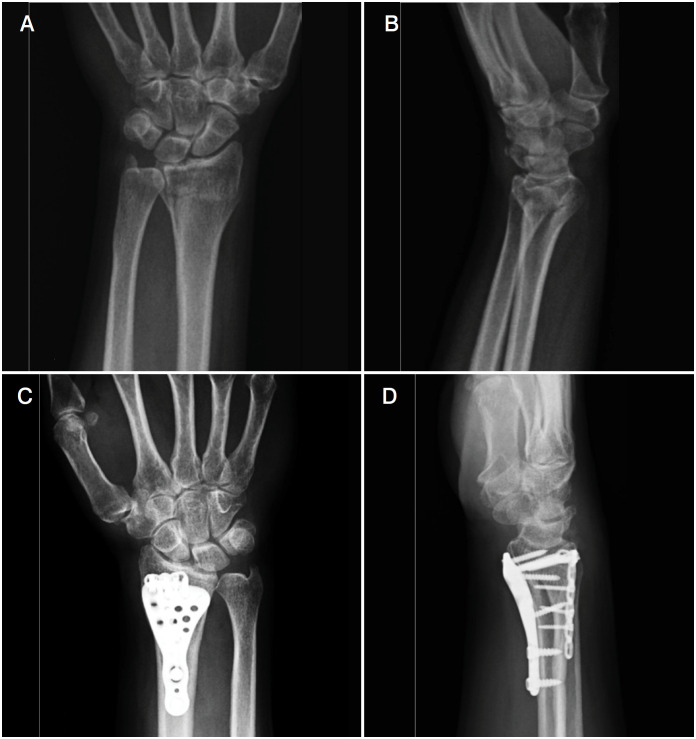
A) Preoperative anteroposterior radiograph showing distal radius fracture, AO 2R3-C3, Melone type 2a. B) Preoperative lateral radiograph showing distal radius fracture, AO 2R3-C3, Melone type 2a. C) X-ray with anteroposterior after fixation with the combined volar-dorsal plates. D) X-ray with lateral after fixation with the combined volar-dorsal plates.

For combined volar-dorsal plate fixation, a volar anatomically locked distal radius plate was placed using the standard volar Henry approach. The insertion of brachioradialis was loosened for radius styloid reduction and to ensure radial inclination. Volar facet fixation was used as a template for other fragments. Then the second incision over Lister's tubercle was used. The extensor retinaculum was opened in the S-shape. Tendons and posterior interosseous nerve were mobilized, and dorsal capsulotomy was performed. Articular fragments were reduced and supported with allograft if needed. Lunate facet and styloid process were fixed with locked 2 mm miniplates. After dorsal plate fixation, the extensor retinaculum tendon was used to cover the dorsal plate. Then, screws of the volar plate for the radial styloid were inserted. Ulnar styloid fractures were not fixed. (Figure 2) Fluoroscopic imaging was used for the assessment of articular reduction. Active and passive wrist and finger motions were started on the postoperative first day. After three weeks, physical therapy was initiated.

### Outcome Measures

Arbeitsgemeinschaftfür Osteosynthesefragen (AO)/Orthopaedic Trauma Association (OTA) and Melone classifications were used for classification of distal radius fractures. All fractures were evaluated with X-ray imaging.

Follow-ups were in one week, three weeks and six weeks, three months, six months, 12 months, and 24 months. Mayo wrist score, DASH (The Disabilities of the Arm, Shoulder, and Hand) questionnaire, and VAS (the Visual Analog Scale) were evaluated for scoring systems. Clinically, the grip strength of the operated side and the opposite side were evaluated. Radiologically, radioulnar deviation arc (radial deviation plus ulnar deviation degree), volar tilt, radial inclination, radial height, ulnar variance, and carpal sag (translation of the carpus with respect to the long axis of the radius) were evaluated.^15-17^

### Statistical Analysis

Statistical analysis was performed using the IBM SPSS for Windows version 25.0 software (IBM Corp., Armonk, NY, USA). Descriptive data were expressed in mean ± standard deviation (SD), range (min-max), or number and frequency, where applicable. The Kolmogorov–Smirnov test was used to determine whether the data were distributed normally. Independent samples t-test was used to compare the groups. Paired samples t-test was used to compare the data within the group. A p-value of p < 0.05 was considered statistically significant.

## RESULTS

According to the Arbeitsgemeinschaftfür Osteosynthesefragen (AO)/ Orthopaedic Trauma Association (OTA) classification, all fractures were 2R3-C3. According to Melone classification, 11 fractures were type 2a (22.4%), 13 were type 2b (26.5%), seven were type 3 (14.3%), and 18 were type 4 (36.7%).^18^ (Table 1) Gender, side, and dominancy were shown in Table 1.

**Table 1:** Comparison of the demographical characteristics and Mayo scores between groups.

		Group 1 n: 23		Group 2 n:26		TOTAL n:49		p-value
		n	%	n	%	n	%	
Gender	Female	8	34.8	12	46.2	20	40.8	0.430
	Male	15	65.2	14	53.8	29	59.2	
‘Side	Right	16	69.6	18	69.2	34	69.4	0.980
	Left	7	30.4	8	30.8	15	30.6	
Melone Classification	Type 2a	9	39.1	2	7.7	11	22.4	0.030*
	Type 2b	5	21.7	8	30.8	13	26.5	
	Type 3	3	13.0	4	15.4	7	14.3	
	Type 4	6	26.1	12	46.2	18	36.7	
Dominance	Dominant side	15	65.2	20	76.9	35	71.4	0.376
	Nondominant side	8	34.8	6	23.1	14	28.6	
Injury Mechanism	Motor vehicle accident	2	8.7	6	23.1	8	16.3	0.006*
	Falling	12	52.2	18	69.2	30	61.2	
	Work accident	5	21.7	2	7.7	7	14.3	
	Sports injury	4	17.4	0	0	4	8.2	
MAYO	Poor	3	13.0	6	23.1	9	18.4	0.391
	Good	7	30.4	10	38.5	17	34.7	
	Satisfactory	6	26.1	2	7.7	8	16.3	
	Excellent	7	30.4	8	30.8	15	30.6	

* Independent samples t-test.

Combined volar-dorsal plates were applied to 23 patients (46.9%) out of 49 patients, and 26 patients (53.1%) were operated on with external fixation. The mean interval between injury and surgery was 4.52±2.12 (range: 1 to 9) days in the combined volar-dorsal plate group and 3.38±1.76 (range: 1-7) days in the external fixation group. There was a statistical difference between two groups. There was a statistical difference between two groups (p=0.047). The mean duration of hospitalization was 5.34±2.28 (range: 3 to 12) days in the combined volar-dorsal plate group and 2.84±0.88 (range: 2 to 4) days in the external fixation group. There was a (p<0.001). The mean follow-up time was 3.17±1.07 (range: 2-5) years in the combined volar-dorsal plate group and 3.76±1.5 (range: 2-6) years in the external fixation group. There was no statistical difference between two groups (p=0.155). (Table 2)

**Table 2:** Functional and radiological results of patients.

	Group 1 n:23	Group 2 n:26	TOTAL n:49	p-value
	Mean (SD)	Mean (SD)	Mean (SD)	
Age	44.00 (12.47)	43.73 (18.48)	43.85 (15.78)	0.952
Time between trauma and surgery (day)	4.52 (2.12)	3.38 (1.76)	3.91 (2.00)	0.047*
Hospitalization time (day)	5.34 (2.28)	2.84 (0.88)	4.02 (2.09)	<0.001*
Operation time (minutes)	103.04 (12.76)	53.46 (11.02)	76.73 (27.62)	<0.001*
Follow-up time (year)	3.17 (1.07)	3.76 (1.50)	3.49 (1.34)	0.115
DASH	10.87 (8.12)	14.92 (9.10)	13.02 (8.81)	0.109
VAS	2.13 (0.96)	2.61 (1.47)	2.38 (1.27)	0.176
Radioulnar deviation arc	41.17 (7.13)	33.46 (9.59)	37.08 (9.29)	0.003*
Flexion angle (degree)	50.00 (14.84)	42.69 (13.80)	46.12 (14.62)	0.081
Extension angle (degree)	53.04 (15.28)	45.38 (13.33)	48.98 (14.64)	0.067
Volar tilt (degree)	8.73 (4.83)	4.15 (9.49)	6.30 (7.94)	0.042*
Radial Inclination (degree)	22.04 (5.18)	18.00 (4.66)	19.89 (5.27)	0.006*
Radial Height (mm)	11.56 (3.5)	12.00 (3.51)	11.79 (3.47)	0.667
Ulnar Variance	1.04 (1.42)	1.00 (1.20)	1.02 (1.29)	0.908
Carpal Sag (mm)	1.07 (0.54)	1.4 (0.55)	1.24 (0.56)	0.047*
Supination-pronation (degree)	147.39 (11.16)	145.92 (11.21)	146.61 (11.10)	0.649

SD: standard deviation.

* Independent Samples t-test.

The mean operation time was 103.04±12.76 (range: 85 to 130) minutes in the combined volar-dorsal plate group and 53.46±11.02 (range: 40-75) minutes in the external fixation group. There was a statistical difference between two groups (pp<0.001). The mean radio-ulnar deviation arc was 41.17°±7.13° (range: 28° to 58°) in combined volar-dorsal plate group and 33.46°±9.59° (range: 20° to 50°) in the external fixation group. There was a statistical difference between two groups (p=0.003). (Table 2)

The mean volar tilt was 8.73°±4.83° (range: 1° to 16°) in combined volar-dorsal plate group and 4.15°±9.49° (range: -20° to 17°) in the external fixation group. There was a statistical difference between two groups (p=0.042). The mean radial inclination was 22.04°±5.18° (range: 11° to 30°) in combined volar-dorsal plate group and 18.00°±4.66° (range: 9° to 26°) in the external fixation group. There was a statistical difference between two groups (p=0.006). The mean carpal sag was 1.07±0.54 (range: 0.3 to 2) mm in the combined volar-dorsal plate group and 1.40±0.55 (range: 0.2 to 2.4) mm in the external fixation group. There was a statistical difference between two groups (p=0.047). (Table 2)

The mean grip strength was 27.87±8.84 (range: 17 to 42) kg and 80% of the opposite side in the combined volar-dorsal plate group. The mean grip strength was 30.84±11.07 (range: 16-47) kg and 87.9% of the opposite side in the external fixation group. There was no statistical difference between two groups (p=0.302). (Table 3) There was no implant irritation, infection, major nerve damage, malunion, non-union, and tendon rupture in combined volar-dorsal plate group. There was complex regional pain syndrome in only three patients. Postoperative swelling and bullous lesions were observed in six patients, which were healed with local wound healing. There was complex regional pain syndrome in six patients in the external fixation group, pin site infection in seven patients, and finger stiffness in three patients. There was no carpal tunnel syndrome, major nerve damage, malunion, non-union, and tendon rupture in external fixation group. A secondary operation was not needed in any patients.

**Table 3:** Grip strength values of patients.

Grip Strength	Group 1 n: 23		Group 2 n: 26		TOTAL n: 49		p-value
	Mean	SD	Mean	SD	Mean	SD	
Operated side	27.87	8.84	30.84	11.07	29.44	10.10	0.302
Opposite side	34.78	8.23	35.07	10.64	34.93	9.49	0.914
p-value	<0.001*		<0.001*		<0.001*		

SD: standard deviation.

* Paired samples t-test.

## DISCUSSION

Joint anatomy and design have gained importance recently. Posttraumatic arthrosis development due to joint stepping and advances in plate technologies have increased the importance of open reduction internal fixation.^19^ External fixation is a well-proven common treatment for intra-articular distal radius fractures. The reduction is provided by the ligamentotaxis method and supported by percutaneous or mini-open procedures. Limited information is available in the literature regarding the combined dorsal-volar plate.^6-8^

Ring et al. [20] evaluated the results of 25 patients operated on with volar - dorsal plates. They stated that all the fractures were healing. The mean ROM was 56 degrees, and they achieved 54 ° extension, 51 ° flexion, 79 ° pronation, and 74 ° supination. The average grip strength was 78% compared to the opposite side. The mean dorsal angulation of the radius was 2 degrees. They obtained a mean radial tilt of 21°, 0.8 mm positive ulnar variance, and 0.7 mm joint displacement. Seven patients developed radiographic signs of arthrosis. According to the Gartland and Werley grading system, it was excellent for 13 patients, good for 11 patients, and moderate for one patient. According to Green and O’Brien criteria, it was evaluated as excellent for five patients, good for five patients, moderate for 14 patients, and poor for one patient. Kibar^6^ found that the VAS score was 2.1, the mean grip strength was 25.2.

In Mayo wrist score, Kibar found five patients had excellent, six of them had good, six patients had satisfactory, and three patients had poor results. Medlock et al.^14^ evaluated the clinical results of 18 patients who were treated for intraarticular distal radius fractures with a volar-dorsal combined plate. They observed the union was achieved in all fractures. Average ROM was measured as 64%. Proper alignment and length were obtained in all patients. The average grip strength was 71% on the opposite side. According to the Modified Green and O’Brien system, they had ten good, seven intermediate, and one bad result, with an average fast DASH score of 29. There was no wound infection, tendon rupture, or major nerve injury. One patient required skin grafting due to a volar wound closure problem. Sagefors et al.^21^ reported the results of 74 patients who operated on combined volar - dorsal plates in their study. Average pronation was 94%, supination 94%, extension 76%, flexion 74%, grip strength 82% relative to the unaffected side. VAS was recorded as 0 at rest and two at the activity. The mean score of the Quickdash was 14.8. The mean Batra score was 88; radial angulation was 21 degrees; volar tilt was 2.5 degrees. None of the patients had tendon rupture or complex regional pain syndrome. In 2 patients, oral therapy was administered due to the infection, and the dorsal plaque was removed due to dorsoradial wrist pain and extensor tenosynovitis in 21 of 74 patients. In our study, all fractures were healed. In mean joint ROM of our patients, flexion was 50, extension was 53, supination-pronation was 147 degrees. Mean grip strength was 80% compared to opposite side. Mean DASH score was 10,87, mean VAS score was 2,13. In Mayo classification, 7 patients were excellent and 6 patients were satisfactory in our study. There was no implant irritation, tendon rupture, infection and major nerve damage in our study. In three patients, there was transient reflex regional pain syndrome, which was improved by conservative treatment. Our findings were consistent with the literature, and complication rates were also lower than the others. Wei et al.^22^ found that external fixation results in better grip strength, wrist flexion and remains a viable surgical alternative compared to ORIF. According to a meta-analysis, Xie et al.^23^ analyzed DASH and grip strength of external fixation in three, six, and 12 months postoperatively. They stated that; DASH scores were between 123 and 147, and grip strengths were between 169 and 271 in approximately 35 studies. And 12-month radiological results were also similar. They stated the patients, who were operated on with external fixation, had minor and major complications, such as finger stiffness, tendon rupture, infection, complex regional pain syndrome, malunion, and non-union. In our study, all fractures were healed. We found flexion as 42 degrees, extension as 45 degrees, and pronation-supination as 145 degrees. DASH score was 14,92, and VAS score was 2,61. In Mayo classification, eight patients were excellent, and two patients were satisfactory. Grip strength was 87% compared to the opposite side. There were six patients with complex regional pain syndrome, seven patients with pin site infection, and three patients with finger stiffness in our study. Roh et al.^24^ stated that there were no significant differences in grip strength, motion, or functional scores between patients operated with the volar plate and external fixation groups at 12 months. And also, the volar plate group showed superior short-term results for functional recovery. In another study, Richard et al.^25^ found that volar plate fixation has an overall decreased incidence of complications and significantly better motion in flexion-extension and supination–pronation than external fixation. Rizzo et al.^26^ evaluated 55 patients with distal radius. They stated that grip and range-of-motion data were similar, DASH scores, frequency of rehabilitation, and some radiographic parameters were superior in patients treated with ORIF. In a prospective study by Greval et al.^27^, the results of 62 patients operated with the dorsal plate and external fixation pinning due to AO type C distal radius fracture were compared. DASH score was not significantly different. There were higher complications in the dorsal plate group. The dorsal plate group also had higher pain levels at one year than the external fixator group; however, the pain level became equal after the plaque was removed. The external fixator group showed an average of 97% grip strength than the normal and 86% in the dorsal plate group. Wang et al.^28^ investigated 895 patients by questioning the patients who were operated on with external fixator nailing or dorsal bridging plate for distal radius fractures. It was reported that the infection rate and complex regional pain syndrome were lower in dorsal bridge coverage. Gartland and Werley scores were better on the dorsal bridge plate. There was no significant difference in the DASH score and radiographic parameters. When we compared external fixation and combined volar-dorsal plate; grip strength, joint ROM, DASH and VAS scores were similar in our study. Radiological parameters were better in combined volar-dorsal plate group. Complication rates were lower in combined volar-dorsal plate group. Since we used the extensor retinaculum tendon to cover it over the plate after dorsal plate fixation, tendon rupture was rare. Rehabilitation time was longer in external fixation group. Infection rate was lower in combined plate group.

Early range of motion exercises and the advantage of mobilization are some advantages of the combined volar-dorsal plate. Complication rates were higher in the external fixation group. In the literature, dorsal plates with thicker profiles resulted in higher complication rates. In our study, we performed dorsal reduction and fixation with plates with a thinner profile. So, we observed lower complication rates with mini-plate for fixation. Operation time was lower in the external fixation group compared to the combined dorsal-volar plate group. But, despite external fixation is a more comfortable procedure, the combined dorsal-volar plate needs a more experienced surgical technique.

The present study had some limitations. Our study had a retrospective design. The patient number was small. Surgical techniques were chosen according to surgeons’ preferences. The number of patients with Type 4 fractures according to the Melone classification was relatively higher in the external fixation group. The follow-up period of our study included the mean follow-up of three years, which is another limitation. A longer-term follow-up of the procedure was required to detect tendon rupture or degeneration.

## CONCLUSION

In conclusion, we found similar functional results in treating intra-articular distal radius fractures with the combined volar-dorsal plate osteosynthesis and external fixation. The combined volar-dorsal plate osteosynthesis could be an alternative to external fixation because they have lower complication rates and permit early mobilization.
